# Amantadine Ameliorates Dopamine-Releasing Deficits and Behavioral Deficits in Rats after Fluid Percussion Injury

**DOI:** 10.1371/journal.pone.0086354

**Published:** 2014-01-30

**Authors:** Eagle Yi-Kung Huang, Pi-Fen Tsui, Tung-Tai Kuo, Jing-Jr. Tsai, Yu-Ching Chou, Hsin-I Ma, Yung-Hsiao Chiang, Yuan-Hao Chen

**Affiliations:** 1 Department of Pharmacology, National Defense Medical Center, Taipei, Taiwan; 2 Graduate Institute of Computer and Communication Engineering, National Taipei University of Technology, Taipei, Taiwan; 3 Department of Neurological Surgery, Tri-Service General Hospital, National Defense Medical Center, Taipei, Taiwan; 4 School of Public Health, National Defense Medical Center, Taipei, Taiwan; 5 Department of Neurosurgery, Taipei Medical University Hospital, the PhD Program for Neural Regenerative Medicine, Graduate Institute of Neural Regenerative Medicine, Taipei Medical University, Taipei, Taiwan; Xi'an Jiaotong Univesity School of Medicine, China

## Abstract

**Aims:**

To investigate the role of dopamine in cognitive and motor learning skill deficits after a traumatic brain injury (TBI), we investigated dopamine release and behavioral changes at a series of time points after fluid percussion injury, and explored the potential of amantadine hydrochloride as a chronic treatment to provide behavioral recovery.

**Materials and Methods:**

In this study, we sequentially investigated dopamine release at the striatum and behavioral changes at 1, 2, 4, 6, and 8 weeks after fluid percussion injury. Rats subjected to 6-Pa cerebral cortical fluid percussion injury were treated by using subcutaneous infusion pumps filled with either saline (sham group) or amantadine hydrochloride, with a releasing rate of 3.6mg/kg/hour for 8 weeks. The dopamine-releasing conditions and metabolism were analyzed sequentially by fast scan cyclic voltammetry (FSCV) and high-pressure liquid chromatography (HPLC). Novel object recognition (NOR) and fixed-speed rotarod (FSRR) behavioral tests were used to determine treatment effects on cognitive and motor deficits after injury.

**Results:**

Sequential dopamine-release deficits were revealed in 6-Pa-fluid-percussion cerebral cortical injured animals. The reuptake rate (*tau* value) of dopamine in injured animals was prolonged, but the *tau* value became close to the value for the control group after amantadine therapy. Cognitive and motor learning impairments were shown evidenced by the NOR and FSRR behavioral tests after injury. Chronic amantadine therapy reversed dopamine-release deficits, and behavioral impairment after fluid percussion injuries were ameliorated in the rats treated by using amantadine-pumping infusion.

**Conclusion:**

Chronic treatment with amantadine hydrochloride can ameliorate dopamine-release deficits as well as cognitive and motor deficits caused by cerebral fluid-percussion injury.

## Introduction

Traumatic brain injury (TBI) is a significant cause of death and disability in industrialized countries. Its severity and its impact on cognitive, learning, and motor functions can vary [1]–[3]. Because of the complexity of injuries and the variety of patient presentations within a TBI population, establishing therapeutic strategies for TBI is challenging.

Anatomic and functional studies have shown that the dopamine system may be vulnerable to TBI, and dopamine suppression after a TBI has been shown in previous studies [4], [5]. In addition, a significant pharmacotherapy that has consistently shown benefits to attention, behavioral outcomes, executive functions, and memory is dopaminergic (DA) therapy [6]–[8], but the aspect of TBI pathology targeted by DA therapy remains unclear. Pharmacological interventions to elucidate cognitive and behavioral deficits in patients with head injuries are now being performed clinically, although empirical studies supporting this practice are limited [9], [10], and the use of psychostimulant drugs (e.g., methylphenidate) for head injuries [11], [12] may indicate that cognitive and learning impairments are related to a deficiency in the dopamine system after head injury. Furthermore, according to a statement in a recent clinical trial of amantadine in treating head injuries that was published in NEJM in 2012 [8], future research should focus on determining the pathophysiological characteristics of patients who responded to amantadine, the most effective dosage, and the duration of treatment and timing of its initiation, as well as the effectiveness of amantadine in patients with brain injuries. In addition, Sawyer et al. reviewed the studies of amantadine (<6 mo after injury) for enhancement of arousal or cognition in patients with TBI by searching through Medline, and they indicated that the results were inconsistent between studies, largely due to variability in design, heterogeneity in patient populations, the amount of time following injury, and the use of numerous different outcome measures. Despite these limitations, improvements in arousal and cognition, as documented by the Glasgow Coma Scale and other measures, have been observed in patients with TBI when amantadine has been initiated between 3 days and 5 months after injury [13]. Thus, although the effect of treatment is beneficial to patient outcomes, the time course of the treatment is still controversial [8], [13]. Nevertheless, ongoing research using animal models has begun to elucidate the pathophysiology of DA alterations after TBI.

Amantadine is an antagonist of the NMDA type glutamate receptor. It facilitates dopamine release, blocks dopamine reuptake, and inhibits microglial activation and neuroinflammation, and it has been the subject of considerable interest and clinical use for patients with prolonged disorders of consciousness after TBI [13], [14]. Amantadine has emerged in the literature as a medication with potential benefits for patients with head injuries and has been used in basic animal studies and clinical trials [8]. Preliminary studies have shown that amantadine hydrochloride accelerates functional recovery during the active treatment of patients with brain injuries [13]–[15]. To date, no studies have explored the potential of amantadine hydrochloride for providing behavioral recovery in chronic treatments. Nor has the mechanism of amantadine therapy been studied by focusing on its effects on bursting and tonic dopamine release after injury, which can provide a direct reversal of dopamine release and result in improved motor functions.

The mechanisms of amantadine are still poorly understood, making the use of animal studies to elucidate its effects a crucial step in enhancing its clinical use. Therefore, in this study, we used chronic amantadine treatment on animals with fluid-percussion-induced injury with pump infusion for 8 weeks and analyzed changes in behavior and tonic as well as bursting dopamine release conditions after injury to determinate the effects of amantadine on dopamine release at the subacute and chronic stages after fluid-percussion injury.

## Materials and Methods

### Animals

Young adult male Sprague-Dawley rats (LASCO Taiwan Co., Ltd., Taiwan) were used, and all procedures were approved by the Animal Care and Use Committee of the National Defense Medical Center (NDMC). Animals were provided food and water ad libitum and were housed in a 12-h light-dark cycle room. The total number of animals used in this study was 130. A total of 65 animals were measured using FSCV, including those that were subjected to sham (control, n = 5), low (1.9±0.2 atm, n = 15), and high (6.0±0.5 atm, n = 15) fluid percussions, as well as those subjected to high percussion injury with amantadine therapy (n = 15) and high percussion injury with saline (n = 15). The total number of animals analyzed using HPLC was 33; n = 15 for both the 2-Pa and 6-Pa-injured groups and n = 3 for the sham group. The number of animals used for the behavioral tests was 32, with n = 9 each for the sham, 6-Pa-injured, and 6-Pa-injured with amantadine therapy groups, and n = 5 for the 6-Pa-injured with saline group. The n values for the FSCV data reflect the number of brain slices used for each time point. The experimental protocol is shown as a diagram in Supplemental Figure S1.

### Fluid Percussion Traumatic Brain Injury

A fluid percussion device (model HPD-1700, Dragonfly R&D, USA) was used to produce TBIs in rats, as described by Matsushita et al. [16]. Injury was induced by striking the piston with a weighted metal pendulum released from a predetermined height. The resulting rapid injection of a small volume of saline into the closed cranial cavity caused a pulse of increased intracranial pressure that was associated with a deformation in the brain. Pressure pulses were measured extracranially with a pressure transducer, recorded on a digital real-time oscilloscope (TDS210, Sony Tektronix Corp., Japan), analyzed by WaveStar software (Sony Tektronix Corp., Japan) and, based on prior instrument calibration, expressed in atmospheres (atm). The fluid percussion device delivered transient pressure fluid pulses in a constant wave form and at a constant duration (17–21 ms) to cause brain injury [17].

### Surgical Preparation and Fluid Percussion Model

Male SD rats (6-week old) weighing 200–250 g were anesthetized with chloral hydrate (4 mg/100 mg). With the animals in a stereotaxic frame, the scalp and temporal muscle were reflected. A 4.8-mm-diameter craniectomy was performed over the right parietal cortex, 3.8 mm posterior to the bregma and 2.5 mm lateral to the midline, taking care not to penetrate the dura [18]. A cranial Leur adapter of 2.5 mm in inner diameter was placed on the craniectomy site and tightly mounted to the skull using dental acrylic resin.

The cranial Leur adapter was filled with saline and attached to the fluid percussion device. Animals were subjected to sham (control, n = 21), low (1.9±0.2 atm, n = 22), or high (6.0±0.5 atm, n = 22) fluid percussions. Survival rates were consistent with those reported by Matsushita et al. (2000) and McIntosh et al. (1989). Procedures using animals were approved by the Ethics Committee for Animal Experiments of the National Defense Medical Center (NDMC).

### Amantadine Infusion Pump Implantation and Therapeutic Protocol

For the amantadine treatment, we performed the pumping infusion implantation subcutaneously on the back of the rats. Mini-osmotic pumps (ALZET ® model 2006, pumping rate: 0.15 µl/h, reservoir volume: 200 µl, DURECT corporation, USA) were implanted 5 d after injury. Amantadine (40 mg) was added to 200 µl 0.9% saline and the solution was delivered into each mini-osmotic pump. The release rate was 0.15 µl/h, and the dose of the amantadine for each rat was 3.6 mg/kg per day. The rats were anesthetized with an intraperitoneal injection of sodium pentobarbital (50 mg/kg), and then the mini-osmotic pumps filled with amantadine were implanted into the subcutaneous layer of the nuchal region of the rat.

### FSCV Recordings

Carbon fibers (7 µm diameter; Goodfellow Corp., Oakdale, PA, USA) were prepared as previously described [19], [20]. Briefly, micropipettes containing the carbon fibers were backfilled with a 4 M potassium acetate/150 mM KCl solution and connected to a standard patch pipette holder/headstage assembly. CHEM-CLAMP (cornerstone series) Voltammeter-Amperometer (Dagan Corporation, USA) was used to change the carbon fiber electrode electrical potential and to measure the current. Voltammetric scans and electrical stimulus timing protocols were performed using PCI-based analog-to-digital boards (National Instruments, Austin, TX, USA) and LabView-based software (courtesy of Dr. Mark Wightman, University of North Carolina, Chapel Hill, NC, USA). During electrochemical detection, the potential of the carbon fiber was driven from −0.4 to 1.0 V and back to −0.4 V using a triangular waveform (400 V/s, 7 ms duration) applied every 100 ms. A 5-s (50-scan) control period was used to obtain a stable background current that was digitally subtracted from that obtained during the peak of the response following electrical tissue stimulation. Peak oxidation currents were converted to DA concentrations using a calibration performed for each electrode with a 1 µM DA standard solution. All signals used in the statistical analyses matched the expected voltammetric profile for DA [21].

### Electrically Evoked DA Signals in Brain Slices

Under stereoscopic magnification, carbon fibers were lowered to a depth of 100 µm into the dorsolateral striatum. A bipolar stimulating electrode was positioned 75–100 µm from the carbon fiber and constant voltage pulses (1–20 Volt, 1 ms duration) were delivered between voltammetric scans to elicit DA release. Responses were obtained every 2 min, and all of those used for analysis were stable throughout the duration of the recordings. For single-pulse experiments, DA uptake was assessed by fitting a single exponential function to the signal decay using a least-squares minimization algorithm (WinWCP; Dr. John Dempster, Strathclyde Institute for Biomedical Sciences, Glasgow, UK; http://spider.science.strath.ac.uk). A *tau* (τ) value was obtained for each recording site by averaging all time constants obtained from each DA signal generated during input-output curves (stimulus intensity vs. DA signal). The first-order rate constant (k or 1/τ) obtained using this approach provided an index of the efficiency (Vmax/Km) of DA clearance mediated by the DAT [19], [22].

To assess the capacity of axon terminals to release DA during burst stimulation, 3 voltammetric signals were obtained at each recording site using a single pulse and 2, 5, and 10 pulses delivered at 25 Hz. After 3 signals at each site were averaged, the difference between the peak DA signal obtained immediately after burst stimulation or single pulses was determined as DAnp - DA1p, where DAnp is the amplitude of the voltammetric signal for n pulses, and DA1p is the amplitude of the voltammetric signal obtained following a single electrical pulse. Data were obtained for each slice before and during drug treatment. The data were fit to a linear regression model (y = mx+b; Prism 5.02; GraphPad, San Diego, CA, USA), where the slope m represents the relative change in DA concentration per pulse [20].

### High Pressure Liquid Chromatography (HPLC)/Electrochemical Detection

Rats from different groups were sacrificed and the brains were rapidly removed. The striatum and NAc were dissected out and frozen immediately on dry ice. On the day of the assay, the dissected tissue was homogenized in 0.1 mM oxalic acid. The homogenates were centrifuged at 15,000 g for 40 min at 4°C. The resulting supernatants were filtered through a 0.22-µm syringe filter (Millipore, Bedford, MA, USA), followed by high-performance liquid chromatography (HPLC) to determine the concentrations of dopamine (DA), 3,4-dihydroxyphenylacetic acid (DOPAC), and homovanillic acid (HVA). The HPLC system was composed of a reverse phase C-18 column (MD-150, RP-C-18, 3 mm, length: 15 cm; ESA Biosciences, Chelmsford, MA, USA) and a high pressure pump (LC-10AD; Shimadzu, Kyoto, Japan); these were connected to an electrochemical detector (ECD) coupled with three electrodes (Coulochem II; ESA Biosciences). The electrode of the guard cell was set to 40 mV, and electrodes 1 and 2 (for detection) were set to 250 and 350 mV, respectively. Under an isocratic condition, the mobile phase solvent (MD-TM; ESA Biosciences) was circulated at a flow rate of 0.5 mL/min in the system. To quantify the sample peaks, each chemical species (DA, DOPAC, and HVA) was compared to the external standards, which were freshly prepared and injected every five-sample runs, as described previously [23]. The turnover rates for dopamine [(DOPAC+HVA)/DA] were calculated to represent the activity of dopamine neurons.

### Behavioral Tests

#### Rotarod test

Rats were trained 3 times/trial for each week using a rotarod machine (Acceler rotarod for rats 7750; Ugo Basile, Comerio-Varese, Italy), with a fixed rate of 4 rpm before the injury. We tested the motor behavior function of the rats 5 times/trial at 24 h before and after injury, and then tests were performed every week after injury for 8 weeks.

#### Novel Object Recognition (NOR) test

The methods of the NOR test were modified from those originally reported by Ennaceur and Delacour [24]. An acrylic box (50×50×50 cm) consisting of four black walls and one white floor was used as the chamber for the NOR tests. The chamber was also equipped with an overhead camera for recording. The tests were performed in an isolated noise-free room. The NOR tests were executed on a day before injury and 1, 2, 4, 6, and 8 weeks after injury. The NOR tests were performed six times to evaluate functional changes in cognition over time. During the first NOR test, familiar objects (F) used in the first and second trials were grey plastic egg-shaped objects, whereas the novel object (N) in the second trial was a white glass cube-shaped object. However, different sets of familiar and novel objects were used in each single NOR test. The five sets of objects used are: a grey plastic short cylinder-shaped object (F) and a white metal pillar-shaped object (N); a white plastic calabash-shaped object (F) and a transparent glass short cylinder-shaped object (N); a white plastic bottle-shaped object (F) and a brown glass bottle-shaped object (N); a transparent glass bottle-shaped object (F) and a red-and-white plastic ball-shaped object (N); a white plastic bowl-shaped object (F) and a golden metal cylinder-shaped object (N). All of the objects were of a certain weight so that they could not be moved by the rats during the tests. Each NOR test consisted of two trials in two days. On the day before the first trial, the animals were placed in the test chamber without any objects for 1 h for habituation. On day 1, the first trial was executed with two familiar objects located at the center of the chamber (16.6 cm and 25 cm from the walls, 16.6 cm between the two objects). On day 2, the second trial was performed in the same chamber but with one familiar object and one novel object placed in the same positions as those of the objects in the first trial. In both trials, the animals were allowed to explore the objects for 5 min. The totaltimes that animals explored the objects within 5 min was recorded and calculated as 100%. The time the animals spent on exploring the familiar and novel objects was recorded and converted to the percentage of the total exploring time. The ratio of (exploring time of novel minus exploring time of familiar)/total time was also calculated for comparison.

### Statistical Analyses

Demographic characteristics were represented in terms of means plus standard error. Statistical analyses of data for the dopamine release input/output curves and behavioral tests were performed using a two-way analysis of variance (ANOVA) followed by a Bonferroni post hoc test for multiple comparisons. A one-way ANOVA and a Bonferroni post hoc test were used to determine changes in tau values for the injured and control groups. Independent t-tests were used to analyze group differences in the HPLC. And for release the probability experiments, we analyzed the group differences by using analysis of covariance (ANCOVA) testing followed by Student-Newman-Keuls (SNK) testing for multiple comparisons. Mixed effects regression analysis for repeated measures was used to evaluate group differences for evoked DA release at the striatum. All statistical tests were two-tailed and were performed using GraphPad Prism 5.02 (GraphPad Scientific, San Diego, CA, USA). A p-value <0.05 was considered significant for all analyses.

## Results

### Cerebral Cortical Fluid Percussion Injury Influences Striatal Dopamine Release

**Figure 1:**
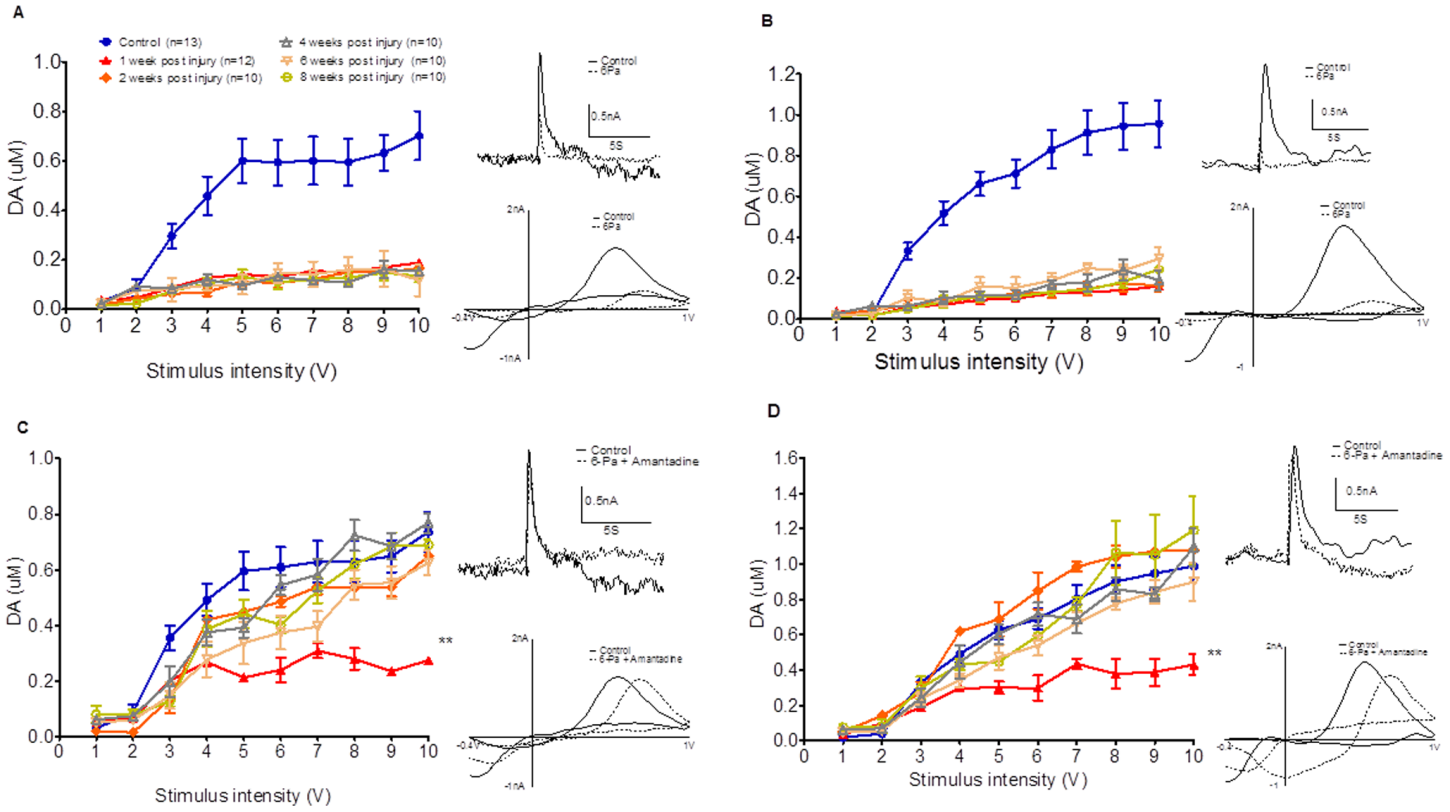
The input/output curves of the evoked dopamine release at 1 (▴, 4 rats, 1 week after injury), 2 (♦, 3 rats, 2weeks after injury), 4 (△, 3 rats, 4 weeks after injury), 6 (□, 3 rats, 6 weeks after injury), and 8 (○, 3 rats, 8 weeks after injury) weeks after injury compared with the control animal group (•) are summarized. Dopamine release was severely suppressed in the fluid percussion injury group under either 1P (A) or 10P (B) stimulation. Amantadine pumping infusion therapy reversed the dopamine-release deficit 2 weeks later (C, solid square **♦**), and the releasing signal even larger than control group under 1 P stimulation (C) at 4 weeks (△, 3 rats, 4 weeks after injury) later and increasing occurred since 2 weeks (**♦**) after injury at 10 pulses-stimulation (D). The inset panels on the right side show representative cyclic voltammetry (CV) trace (upper) and dopamine signals (lower) (A and B: Control (solid line) vs. 6-Pa group (dotted line) at 8 weeks post injury; C and D: Control (solid line) vs. 6-Pa with amantadine therapy (dotted line) at 8 weeks post injury). (Note: *indicates p<0.05; **indicates p<0.01; and ***indicates p<0.001).

The input/output curves of the evoked dopamine release in the striatum at 1, 2, 4, 6, and 8 weeks after 6-Pa injury compared with the control animal group are summarized and shown in Fig. 1. Specifically, release after a single pulse (1 pulse/25 Hz), which mimics tonic dopamine release, is shown in Fig. 1A (F _45,530_ = 6.013 (p<0.001***) of two-way ANOVA followed by Bonferroni posttests, all p<0.001***, in control vs. post-injury groups at 1, 2, 4, 6, and 8 weeks from 3 to 10 stimulus intensity (V)), and release after 10 pulses (10 pulses/25 Hz stimulation), which mimics phasic bursting release, is shown in Fig. 1B (F _45,392_ = 5.397 (p<0.001) of two-way ANOVA followed by Bonferroni posttests, all p<0.001***, in control vs. post-injury groups at 1, 2, 4, 6, 8 weeks from 4 to 10 stimulus intensity (V)). The representative voltammetric signals evoked by a 5V stimulation intensity, obtained from the control and injured animals at different time points are shown in the supplementary data Figs. S2A and C (IT curve), whereas Figs. S2B and D show cyclic voltammograms obtained at the peak of the current consistent with DA in each case (CV curve). The input-output curves for both tonic (1-pulse) (Fig. 1A) and burst firing (10-pulse) (Fig. 1B) at 25 Hz stimulation of dopamine release, elicited in brain slices obtained from control and injured animals, showed that dopamine release decreased and persisted to 8 weeks after injury in the 6-Pa group for the duration of the study.

**Figure 2:**
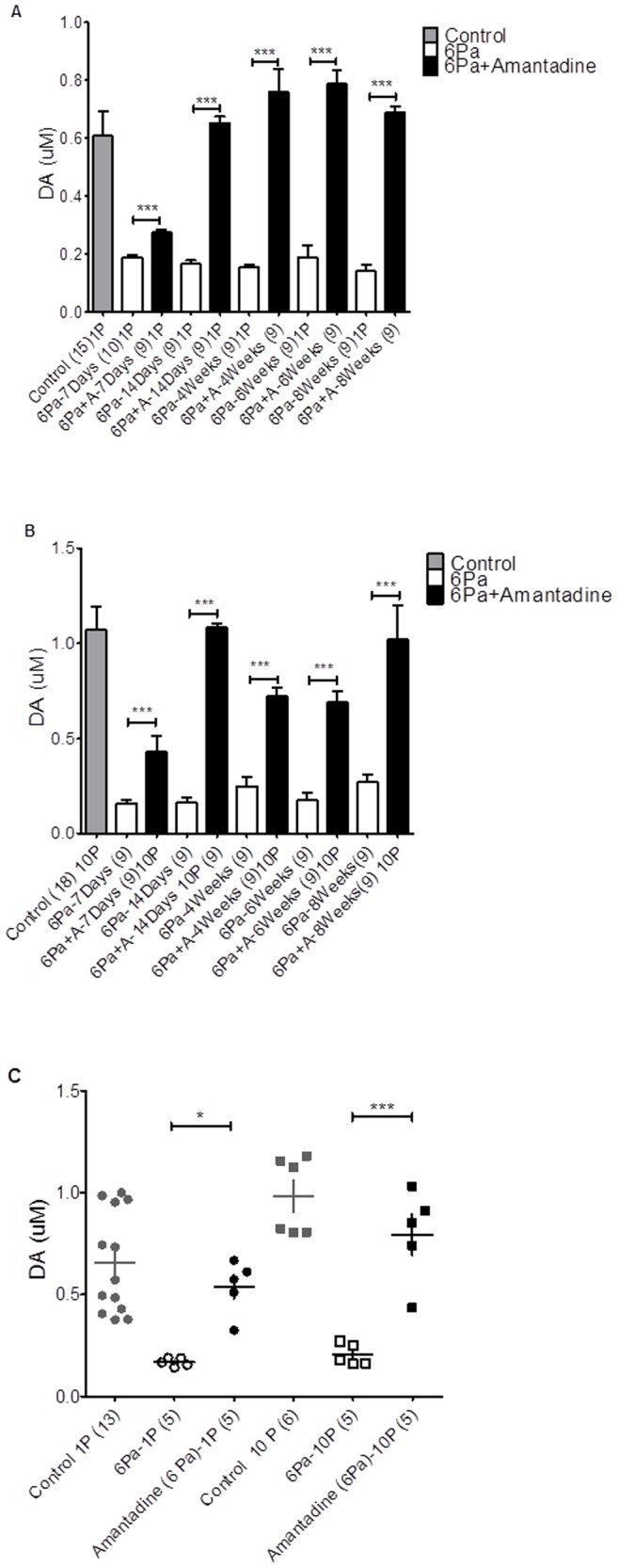
Dopamine signaling values in control and injured animals. The mean values of dopamine signal evoked by 1 Pulse/25 Hz, 10V stimulation intensity of the 6-Pa-injured group is plotted in panel A, and by 10 Pulses/25 Hz, 10V stimulation intensity of 6-Pa injury group is plotted in panel B. There is a significant suppression in injured animals (open box bar) in each time point compared with the control group (gray bar) (p<0.05*, t-test). In the amantadine therapy group (black bar), the signal decreased initially 1 week later and increased 2 weeks after injury. The maximum value of the signal of dopamine release at each time point after injury in the control (solid circle), 6-Pa-injured (open circle), and amantadine therapy (open square) groups are plotted in panel C, which showed that the significant increase in amantadine therapy animal while comparing with 6-Pa injured animal; and there was no significant difference between control and amantadine therapy group. (Note: *indicates p<0.05; **indicates p<0.01; and ***indicates p<0.001).

Compared with the control animals (gray bar in Fig. 2A), significant suppression of the tonic dopamine signals with maximal stimulation intensity (10V) at different time points after injury that are summarized graphically in open bar in Fig. 2A. The dopamine signal for both single- and 10-pulse stimulation was also markedly reduced in the 6-Pa group, compared with the control animals (gray bar in Fig. 2B), and the reduction persisted for at least 8 weeks. The value of the maximum (10V) stimulation of tonic and bursting dopamine release in the injured animals are shown in Figure 2B, which indicates that the signals were suppressed after injury until 8 weeks later.

### Amantadine Restores the Tonic and Phasic Release of Dopamine in Animals with Severe (6-Pa-injured) Fluid-percussion Injury

To investigate the mechanism and therapeutic effect of amantadine on TBI, we performed chronic amantadine therapy using a subcutaneous pumping infusion at 2 d after fluid-percussion-induced injury. The input/output curve shifted close to the control group curve as of 2 weeks after injury and even higher than the control group at 4 weeks post-injury, which indicates that both tonic (single pulse stimulation, Fig. 1C, F_45,298_ = 2.263 (p<0.001***) of two-way ANOVA followed by Bonferroni post-tests, p<0.001***, in fluid percussion injury (FPI) with amantadine therapy vs. control groups under 10 stimulus intensity at 1 week post-injury (V). However, all p>0.05 in FPI with amantadine therapy vs. control under 10V stimulation intensity at 2, 4, 6, and 8 weeks post-injury) and bursting (10-pulse stimulation, Fig. 1D, F_45,335_ = 2.144 (p<0.001***) of two-way ANOVA followed by Bonferroni posttests, p<0.001, in FPI with amantadine vs. control under 10V stimulus intensity at 1 week post-injury. However, p>0.05 in FPI with amantadine vs. control group under 10V stimulation intensity at 2, 4, 6, and 8 weeks post-injury) dopamine release from the brain slice improved after amantadine chronic therapy. Then, while the animals were treated with chronic amantadine infusion after 6-Pa injury, the maximum values (induced by 10V stimulation) of tonic (6-Pa+amantadine group, black bar in Fig. 2A) and burst firing of dopamine release (6-Pa+amantadine group, black bar in Fig. 2B) in the striatal brain slices were analyzed. Compared with the injured animals (6-Pa-injured), chronic amantadine treatment could increase the mean value of dopamine release under 10V/25 Hz stimulation 7 days after injury (black bar in Fig. 2A tonic release, F _10,85_ = 43.06 (p<0.001***) of one-way ANOVA followed by Bonferroni posttests, all p<0.001, in 6-Pa vs. 6-Pa+ amantadine, tonic release at 1, 2, 4, 6, and 8 weeks and burst release in 2B, F_10,70_ = 17.74 (p<0.001) of one-way ANOVA followed by Bonferroni posttests, all p<0.001***, in 6-Pa vs. 6-Pa+ amantadine burst releasing at 1, 2, 4, 6, and 8 weeks), and persisted in increasing the value until week 8 of our period of observation.

The mean maximum values of tonic and burst firing dopamine release at each subsequent time for each group were plotted (Fig. 2C), which shows the significant increase in the amantadine therapy animals while compared with the 6-Pa-injured animals. Furthermore, there was no significant difference between the control and amantadine therapy groups. (In the tonic release (1P) condition, the F_2,20_ = 10.36 (p<0.001***) of one-way ANOVA followed by Bonferroni post hoc test, 6-Pa (1P) vs. Amantadine (6-Pa, 1P), p<0.05*, Control (1P) vs. 6-Pa (1P), p<0.001***, Control (1P) vs. Amantadine (6-Pa, 1P 1P), non-signifcant. Moreover, in the bursting release (10P) condition, the F _2,13_ = 28.81 (p<0.001***) of one-way ANOVA followed by Bonferroni post hoc test, 6-Pa (10P) vs. Amantadine (6-Pa, 10P), p<0.001***; Control (10P) vs. 6-Pa (10P), p<0.001***; and Control (10P) vs. Amantadine (6-Pa, 10P), non-signifcant).

### The Reuptake System of Dopamine was Affected by the Head Injury; the Tau Value was Prolonged by the Head Injury Especially at the Subacute Stage in Either the Mild (2-Pa) or Severe (6-Pa) Group

The clearance rate of dopamine in the striatum was analyzed by comparing decay time constants (*tau*) after cortical injury. Figure 5 shows a variety of decay time constants (*tau*) after injury in different injury groups. A *tau* (τ) value was obtained for each recording site by averaging all time constants obtained from each DA signal generated during input-output curves (stimulus intensity vs. DA signal). The first-order rate constant (k or 1/τ) obtained using this approach provides an index of the efficiency (Vmax/Km) of DA clearance mediated by the DAT. Compared with that of the control animal group, the clearance rate of dopamine in the 6-Pa injury groups was prolonged (decreased) at 1, 2, 4, and 6 weeks after injury (Fig. 5 tonic dopamine release in A and bursting release in B control: gray bar, injured animal: open bar, unpaired t-test, p<0.05*). However, the time constant became shorter than the control values 8 weeks later (p<0.05*), which may indicate that the dopamine clearance rate in the striatum increased at this stage.

Then, we compared the clearance rate of injured animals with the amantadine treated group (6-Pa-injured+amantadine). Our data shows that the clearance rate in the amantadine treated group increased significantly initially (1 week after injury) and then returned to close to the control range at 2, 4, 6, and 8 weeks (black bar in Fig. 5A tonic release and 5B bursting release).

The reduction in DA release observed with single pulse stimulation and FSCV is consistent with the lower levels of evoked DA release in injured animals. However, DA release dynamics differ according to tonic (4 Hz) or phasic (25 Hz) firing patterns of midbrain DA neurons, and these patterns of activity are important for motivated behavior [25], [26]. Therefore, we also evaluated DA release by comparing peak concentrations elicited by single and multiple stimuli delivered at 25 Hz (see Materials and Methods) at 8 weeks after injury. There was a linear increase in DA concentration as a function of pulse numbers (Figs. 5D and E). 6-Pa-injured rat striatal slices showed a significant reduction in DA concentration per pulse, compared to the control animals. Then, amantadine treatment could reverse the dopamine release probability of 6-Pa-injured animals to a level close to that of the control group (Fig. 5D, control rat slope: 38.0+4.6 nM/pulse (blue solid circle), 6-Pa-injured rat slope: 19.2+6.3 nM/pulse (red soild square) and 6-Pa+amantadine slope: 47.0±6.8 nM/pulse (gray open triangle), F = 4.550 (p = 0.021) of ANCOVA followed by SNK for multiple comparisons, control vs. 6-Pa-injured animals, p = 0.042*; control vs. 6-Pa+amantadine, p = 0.527; 6-Pa-injured animals vs. 6-Pa+amantadine animals, p = 0.007**). To determine the role of uptake in the regulation of frequency-dependent DA release in the control and 6-Pa-injured rats, the DAT inhibitor nomifensine was used. Nomifensine (5 µM) infusion tended to increase the frequency-dependent DA signal in the striata of 6-Pa-injured animals but not in those of amantadine treated animals (Fig. 5E, the slope for nomifensine infusion, control: 101.5±2.5 nM/pulse, 6-Pa: 169.2±2.3 nM/pulse; and 6-Pa injury with amantadine: 121.4±1.8 nM/pulse, F = 1.946 (p = 0.159) of ANCOVA followed by SNK for multiple comparisons, control vs. 6-Pa-injured animal, p = 0.058; control vs. 6-Pa+amantadine, p = 0.521, 6-Pa-injured animals vs. 6-Pa+amantadine, p = 0.375). Taken together, these data suggest that DAT significantly limits the size of the electrically released DA signal in striatal slices from control rats and underscores the extent of the decrease in DA release in post-6-Pa-injured rats at 8 weeks that was reversed by chronic amantadine treatment.

To summarize, dopamine reuptake in the striatum was affected by the fluid percussion injury in the acute (within 1 week after injury) and subacute (1 to 2 weeks after injury) stages. The shortening of the *tau* value in the 6-Pa group at 8 weeks suggests that some recovery of the dopaminergic terminals in the striatum at this chronic stage occurs even with severe injury.

### The HPLC Show a Significantly High Metabolic Rate of Dopamine in Severely Injured Animals

**Figure 3:**
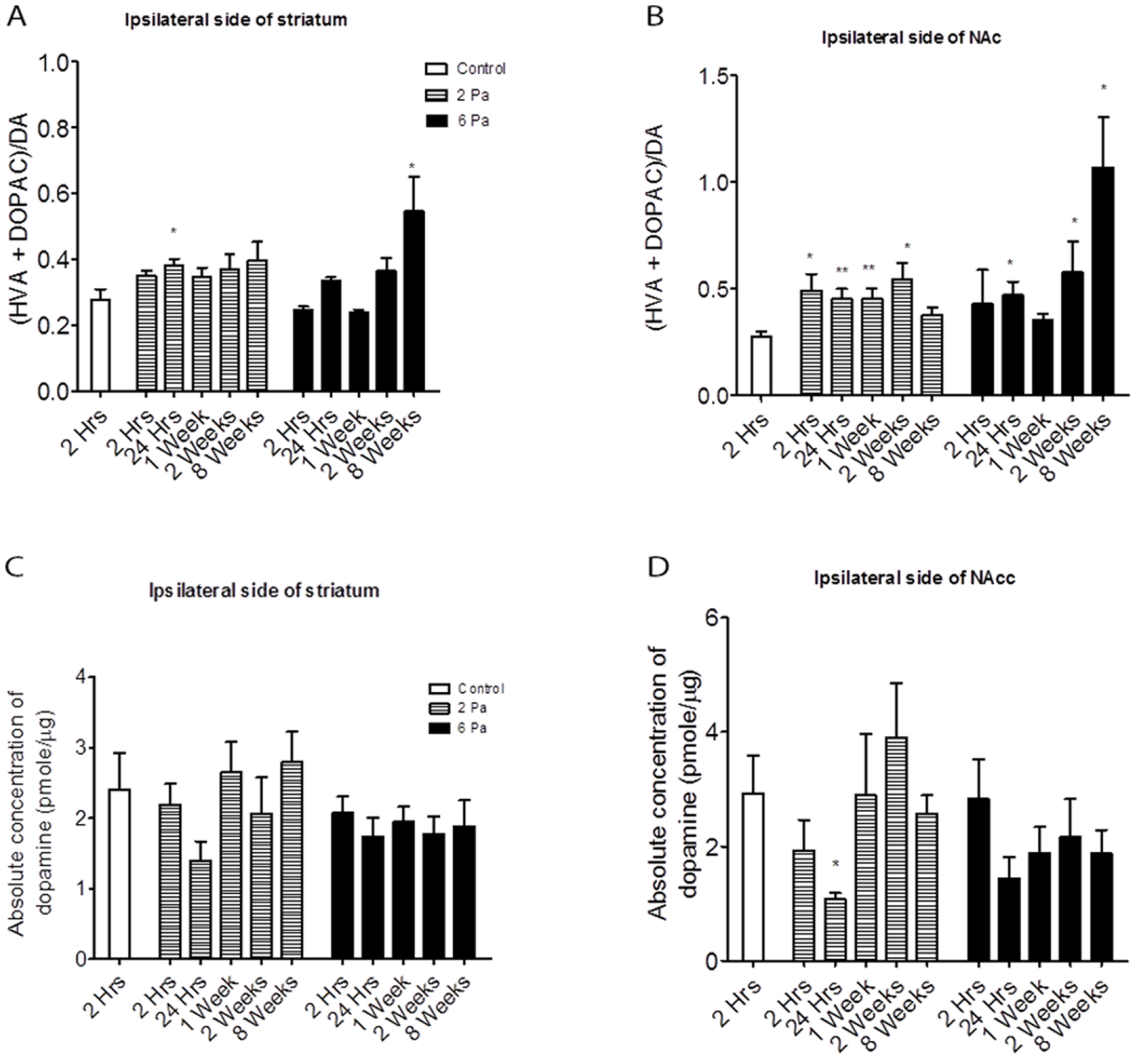
The HPLC surveyed the dopamine turnover rate in the injured animals. (A) The turnover rate of dopamine in the 2-Pa group showed no significant changes (except at 24 hours after injury), but the rate decreased initially in the 6-Pa-injured group and then increased after 8 weeks (p<0.05*). (B) The dopamine turnover rate increased on the ipsilateral side of the nucleus accumbens (NAc) in the 2-Pa injured group and increased significantly at the chronic stage of injury (8 weeks later). The dopamine concentrations in the striatum (C) and the nucleus accumbens (Nac) (D) did not show significant changes after injury and a significant decrease in the Nac of the 2-Pa-injured group was only shown at 1 day post injury (D, unpaired t-test, p<0.05). (Note: *indicates p<0.05; **indicates p<0.01; and ***indicates p<0.001).

Several studies have demonstrated alterations in the dopamine (DA) system after TBI. Therefore, to verify the dopamine metabolism rate after injury, we analyzed the responses of the dopaminergic neurotransmitter systems to fluid percussion injury by testing the concentration of dopamine and its metabolite DOPAC from striatum extracts. In this study, we investigated the temporal changes in DA tissue levels and metabolism at 2-h, 1, 7, 14, and 56 (8 weeks) d after fluid percussion injury or sham injury in rats. DA, VPA, and DOPAC levels were measured by HPLC in the dopamine system. [5]. The turnover rate of striatal dopamine tended to decrease initially in the 6-Pa-injured group and then increase at 8 weeks post-injury (p<0.05*) (Fig. 3A). The dopamine turnover rate tended to increase in the nucleus accumbens (NAc) initially (24 hours and 2 weeks post injury, p<0.05*) in the 6-Pa-injured group and increase significantly at the chronic stage of injury when compared with the mean control group value (Fig. 3B; at 8 weeks post injury, p<0.05*). However, the tissue dopamine concentrations in the striatum (Fig. 3C) and nucleus accumbens (Nac) (Fig. 3D) did not show significant changes after injury, and only showed a significant decrease in the Nac of the 2-Pa-injured group at 1 day post injury (Fig. 3D, unpaired t-test p<0.05*). Furthermore, the HPLC data show significant changes in dopamine turnover at 8 weeks in the 6-Pa group.

### Amantadine Ameliorates Cognitive and Motor Deficits in FPI Animals

**Figure 4:**
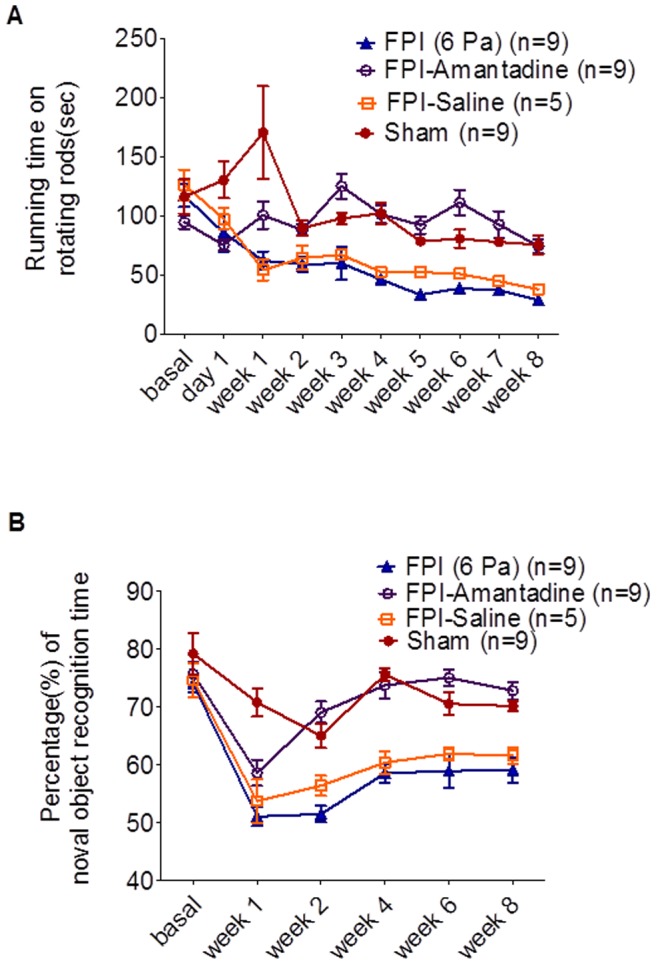
Behavioral test results for the different rat groups. (A) Impairment of rotational behavior was improved after FPI in the FPI-6-Pa+ amantadine group. Compared with the sham group (solid circle, n = 9), there were significant impairments as of one week after injury in the FPI-6-Pa-injured group (injury only, solid triangle; injury with saline treatment, open box; for each group n = 9). These impairments could then be reversed by chronic amantadine treatment of the 6-Pa injury group (n = 9). The running time of the rotarod test for the FPI with amantadine group did not show a significant difference when compared with the FPI only or FPI with saline group at one week post-injury, but an increasingly significant difference from 2 to 8 weeks post-injury was exhibited. Data are presented as mean ±S.E.M and were analyzed via two-way ANOVA followed by Bonferroni posttest, with the F_27,252_ = 3.119, all p<0.05* in the 6-Pa injury vs. 6-Pa injury with amantadine groups and the 6-Pa injury with saline vs. 6-Pa injury with amantadine groups at weeks 2, 3, 4, 5, 6, 7, and 8 post-injury. (B) Impairment of novel object recognition after 6-Pa injury was improved in the 6-Pa injury+amantadine group. In the 6-Pa- injured group (injury only, solid triangle; injury with saline treatment, open box; for each group n = 9), the NOR deficit occurred as of one week after injury, but these deficits could be reversed as of 2 weeks a in the 6-Pa injury+Amantadine group (open circle, n = 9). Data are presented as mean ±S.E.M. The percentage of novel object recognition time in the 6-Pa injury with amantadine treatment group did not show significant abnormality initially, i.e., at one week, when compared with the 6-Pa injury only or 6-Pa injury with saline treatment group, but the percentage increased significantly as of two weeks post-injury and persisted through eight weeks post-injury. The data were analyzed using a two-way ANOVA followed by Bonferroni posttests, with the F_15,158_ = 3.098, all p<0.05* in the 6-Pa injury vs. 6-Pa injury with amantadine and 6-Pa injury with saline vs. 6-Pa injury with amantadine groups at eight weeks post-injury. There was no significant difference (p>0.05) between the FPI with amantadine and the sham groups at eight weeks post-injury. (Note: * indicates p<0.05; **indicates p<0.01; and ***indicates p<0.001).

Cognitive deficit and motor learning impairment also occurred after the injury. The motor learning ability of rats was tested by a rotarod test (Fig. 4A), which showed severe impairment in injured animals 1 week after injury, persisting to 8 weeks later. These impairments could then be reversed by chronic amantadine treatment of the 6-Pa injury group (n = 9). The data are presented as mean ± S.E.M. The running time of the rotarod test for the 6-Pa injury with amantadine group did not show a significant difference when compared with the 6-Pa injury only or 6-Pa injury with saline group at one week post-injury, but an increasingly significant difference from two through eight weeks post-injury was exhibited. F _27,252_ = 3.119 (p<0.001***) for the two-way ANOVA followed by Bonferroni posttests, all p<0.05*, in the 6-Pa injury vs. 6-Pa injury with amantadine and 6-Pa injury -saline vs. 6-Pa injury with amantadine groups at weeks 2, 3, 4, 5, 6, 7, and 8 post-injury. The cognitive function of rats after fluid-percussion-induced injury was surveyed using a NOR test, which showed a low discrimination index (DI) in the injured group. Then, the amantadine treated group showed a better DI 2 weeks later, continuing to 8 weeks after injury. As shown in Fig. 4B, the NOR deficit occurred as of one week after injury, but these deficits could be reversed as of 2 weeks in the 6-Pa injury+amantadine group (open circle, n = 9). Data are presented as mean ±S.E.M. The percentage of novel object recognition time in the 6-Pa injury with amantadine treatment group did not show significant abnormality initially, i.e., at one week, when compared with the 6-Pa injury only or 6-Pa injury with saline treatment group, but the percentage increased significantly as of two weeks post-injury and persisted through eight weeks post-injury. The data were analyzed using a two-way ANOVA followed by Bonferroni posttests, with the F_15,158_ = 3.098, all p<0.05 in the 6-Pa injury vs. 6-Pa injury with amantadine and 6-Pa injury with saline vs. 6-Pa injury with amantadine groups at 8 weeks post-injury. There was no significant difference (p>0.05) between the 6-Pa injury with amantadine and the sham groups at 8 weeks post-injury.

**Figure 5:**
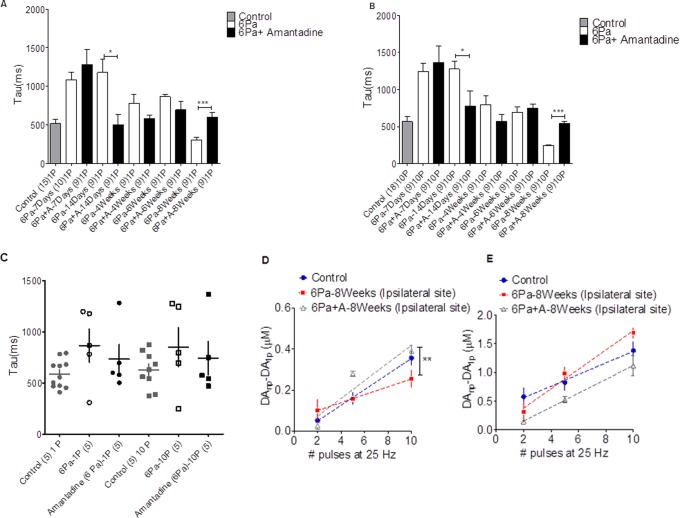
Decay time constants (*tau*) after injury in different injury groups. The uptake efficiency revealed by the *tau* value of tonic (1-pulse evoked) dopamine 6-Pa-injured group shown in panel A indicates prolonged values at 1 and 2 weeks (***P<0.001) after injury, but then reduced values at 8 weeks (p<0.05*) after injury. Panel B shows the *tau* value of the bursting (10-pulse evoked) dopamine release of 6-Pa-injured group with amantadine treatment. A significant prolonged value occurred at 1–2 weeks after injury and then back to normal range after 4 weeks. DA release by comparing peak concentrations elicited by single and multiple stimuli, delivered at 25 Hz (see Materials and Methods) at 8 weeks after injury. The distribution of the mean value of *tau* at each following time point was plotted (Fig. C). The plot shows a linear increase in DA concentration as a function of pulse number (D and E). 6-Pa-injured rat striatal slices demonstrated a significant reduction in DA concentration per pulse, relative to the control animals (D, injured rat slope: 19.2±6.3 nM/pulse vs. control rat slope: 42.9±5.3 nM/pulse, n = 3; p*<*0.05*, Tukey’s *post hoc*). Amantadine treatment reversed the dopamine release probability (D control: solid circle, 6-Pa: open square, 6-Pa+amantadine: open triangle). Nomifensine was used to determine the role of uptake in the regulation of frequency-dependent DA release in the control and the 6-Pa-injured rats. Nomifensine (5 µM) significantly increased the frequency-dependent DA signal in the striatum of both the 6-Pa-injured and amantadine treated groups and higher than control rats (E).

### Amantadine Increases Extracellular DA Levels in the Striatum by Inhibiting the Re-uptake of DA and is Associated with N-methyl-D-Aspartate (NMDA) Receptor

**Figure 6:**
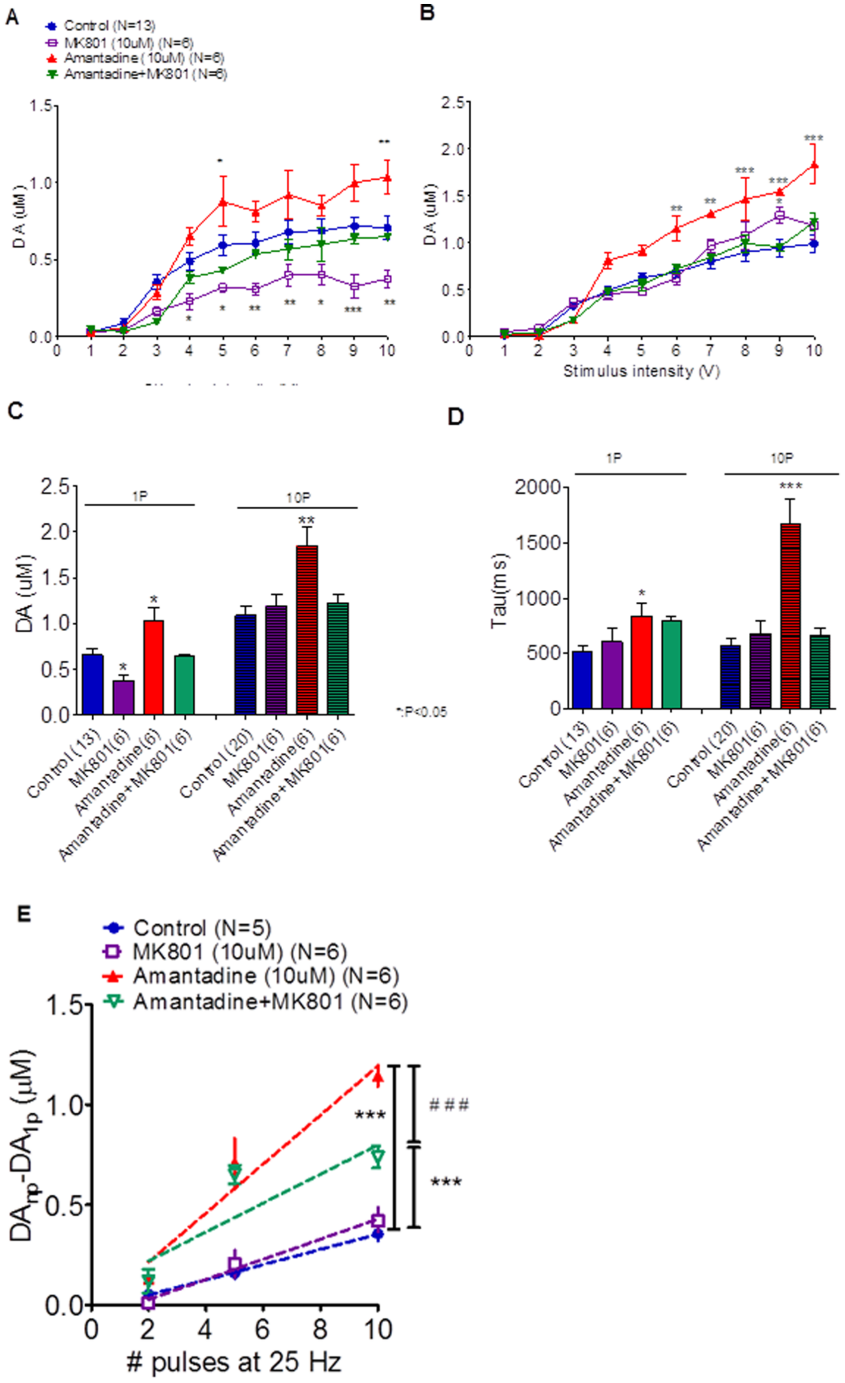
Investigation of the role of NMDA receptors in the effects of amantadine on dopamine release (A and B) showed the I/O curves of the dopamine released in tonic and bursting release states and (C) showed that the maximum value of dopamine release for tonic and bursting release occurred under 10V stimulation intensity. The MK 801 may have a particular impct on the amantadine effect in the tonic release state (Fig. 6A and C, 1-P data) without having much effect in the bursting release state (Fig. 6B and C 10-P data). Then MK-801 would shorten the prolonged effect of the amantadine in dopamine reuptake (Fig. 6D). Then amantadine increased the releasing probability of dopamine and the effect would be suppressed by MK-801, while MK-801 alone did not affect the releasing probability (Fig. 6E, comparing the Slope of each group: control vs. MK-801: p = 0.7054, control vs. amantadine: p<0.001***, control vs. amantadine +MK-801: p = 0.0009***, amantadine vs. amantadine+ MK-801: p<0.001 ^###^). (Note: * indicates p<0.05; ** indicates p<0.01; and *** indicates p<0.001).

To investigate the role of NMDA receptors in the effects of amantadine on dopamine release, we performed additional experiments to survey dopamine release under MK-801 treatment, amantadine treatment, and amantadine with MK-801 treatment. The data shown in Fig. 6A and Fig. 6B indicate that the I/O curves for the dopamine released in tonic and bursting release states were increased by amantadine infusion (A, tonic release: two-way ANOVA analysis, F_27, 235_ = 2.689, followed by Bonferroni post hoc test, control vs. MK-801: t = 3.851, p<0.01** at 10 volts stimulation, control vs. amantadine: t = 3.455, p<0.01** at 10 volts stimulation, control vs. amantadine +MK-801: t = 0.8852, p>0.05 at 10 volts stimulation; B, bursting release: two-way ANOVA analysis, F_27, 277_ = 2.171, control vs. MK-801: t = 1.648, p>0.05, Control vs. amantadine: all p<0.01** since 6 volts stimulation to 10 volts stimulation intensity, control vs. amantadine+MK-801: t = 1.699, p>0.05 at 10 volt simulation intensity), while Fig. 6C shows that the maximum value of dopamine release for tonic and bursting release occurred under 10V stimulation intensity. The MK 801 may have a particular impact on the amantadine effect in the tonic release state (Fig. 6A and C, 1-P data, control vs. MK-801, p<0.05*) without having much effect in the bursting release state (Fig. 6B and C, 10-P data, control vs. MK-801, p<0.05*). The reuptake of dopamine was prolonged by amantadine infusion (Fig. 6D red bar, tau value of control vs. amantadine, p<0.05* in 1P stimulation and p<0.001*** in 10P stimulation). Then MK-801 would shorten the prolonged effect of the amantadine on dopamine reuptake (Fig. 6D, green bar, both tau values in 1P and 10P stimulation in control vs. amantadine +MK-801, p>0.05). Then amantadine increased the releasing probability of dopamine and this effect was suppressed by MK-801, while MK-801 alone did not affect the releasing probability significantly (Fig. 6E, Slope of: control: 27.17±4.88 nM/pulse, MK-801∶28.65±7.18 nM/pulse, amantadine: 87.55±15.72 nM/pulse, amantadine +MK-801∶37.06±10.75 nM/pulse, control vs. MK-801: p = 0.7054, control vs. amantadine: p<0.001***, control vs. amantadine +MK-801: p<0.001***). Our data indicated that amantadine increases extracellular DA levels in the striatum by inhibiting the re-uptake of DA and mediate N-methyl-D-aspartate (NMDA) receptor. When applied more specific NMDA receptor blocker, MK-801, then the increment dopamine releasing effect of amantadine would be suppressed (Fig. 6E, slope comparing; amantadine vs. amantadine+ MK-801: p<0.001 ^###^).

## Discussion

The role of dopamine in cognitive function [27], [28] and the sequelae of TBI cannot be overemphasized [4], [29]. In this study, we chose the striatum as a target to evaluate dopamine release and cognitive/motoric impairment after TBI. Dysfunctional nigrostriatal signaling has implications for cognitive functions, including memory, executive function, and attention, which has been epitomized by investigations in Parkinson’s disease (PD) [30], [31]. Studies have also demonstrated that both the striatum and dorsolateral prefrontal cortex (DLPFC), another DA target, are critical to executive function and working memory [32]. Furthermore, the hippocampus, which is also critical for cognitive function, does not have a high level of DA receptor expression, but it depends on DA activity to modulate its function [33], [34].

The purpose of this study is to investigate chronic DAergic therapies and their effect on behavioral deficits after TBI, and in turn, elucidate the importance of DA for cognitive function/dysfunction after TBI as well as highlight the mechanism of amantadine therapy in TBI.

Our data indicate that both tonic and bursting dopamine release suppression were found in the acute stage (1 week post-injury) and persisted until the chronic stage (8 weeks post-injury) in animals receiving severe cerebral cortical fluid percussion (6-Pa) injuries (Figs. 1A and B), which is consistent with previous reports [4], [35]. These fluctuations of dopamine release may have resulted from the following:

First, DA is known to possess excitotoxic properties [36], and DAergic fibers have been shown to modulate striatal glutamatergic excitotoxicity [37]. The initial increases in DA observed post-TBI in the ultra-early stage may precipitate excitotoxic disruption and, combined with injury-induced oxidative damage to DAergic cellular function, that leads to alterations in DA kinetics and decreased evoked DA release at later time points.

Second, in addition to documented biochemical alterations in DA signaling following TBI, there remains the possibility of structural changes. TBI is known to cause diffuse white matter injury and significant axonal disruptions throughout the CNS (Smith et al., 2003), which may be related to dopaminergic terminal decrease after TBI. Our data show that decreasing may along with the severity of TBI, moderate decreasing and recovered in the mild injured animal (2-Pa group) at the acute and subacute stages, but the decrease still persisted until 8 weeks later in the severe injured (6-Pa) group.

Third, dopamine levels depend on both synthesis and degradation. The deficit in the striatal TH activity 1 week (subacute) and 4 weeks (chronic) after TBI in rats have been reported [29]. Our data for the TH activity assay for per19TH showed compatible results and a significant decrease at day 1 (acute stage) to 2 weeks (subacute stage) (unpublished). Because TH is a rate-limiting enzyme in dopamine synthesis, the decrease in its activity suggests a dopamine synthesis deficit. Increases in TH staining in both the PFC and striatum may represent compensatory regrowth of DAergic fibers that occur as a consequence of DA synapse or axonal pathology that occur following TBI [30]. The regrowth or collateral sprouting of catecholaminergic axons has already been demonstrated in experimentally induced lesions in adult CNS neurons [38]. The spontaneous regrowth of DAergic fibers after partial nigrostriatal denervation caused by TBI has already been observed [39] The TBI-induced expression of TH in the nigrostriatal system might share similar mechanisms.

Fourth, dopamine levels are regulated partially by the trafficking of transporters to and from the cell surface. The functional uptake assay was influenced by dopamine transporters that expressed on the cell surface, whereas western blotting detects all transporter proteins. Dopamine transporters in the striatum are particularly resilient and able to continue functioning normally despite changes in the surrounding circuitry and neuronal tissue [40]. Furthermore, the dopamine transporter is functionally impaired by oxygen radicals after TBI [41]. Markers of oxidative damage have been measured after experimental TBI, with increases at early time points usually returning to normal levels within 72 h after the insult [42], [43]. The time constant prolongation could have resulted from terminal decreasing and then recovered because of terminal regrowth after injury in these animals.

Fifth, TBI may not only cause dysfunction of dopamine release, concentration, and metabolism; but also cause alterations in DARPP-32 phosphorylation as well as a number of crucial intracellular signaling molecules. These alterations cause implications for the function of medium spiny neurons in the striatum and represent another possible level of DA dysfunction following TBI [44].

Dopamine release suppression was reversed by chronic amantadine therapy after fluid percussion injury. The tonic release of dopamine was reversed by the amantadine treatment 2 weeks after fluid percussion injury (Fig. 1C), and the mean value of each subsequent time point under maximum stimulation (10V) was plotted. As shown in Fig. 2A, the dopamine release values under amantadine treatment were higher than those of the injured animals without amantadine treatment. The same situation was observed in the input/output curve of burst firing release of dopamine, which was reversed by the amantadine therapy (Fig. 1D). The mean values of 10V stimulation in amantadine treatment were higher than those of the injured animals alone (Fig. 2B).

HPLC showed an increase in the metabolism rate of dopamine and decrease in the dopamine level in the chronic stage. Variations in dopamine levels after TBI have been shown in previous reports and are shown in our data as well, but these data seem inconsistent and controversial as they may indicate alterations in dopamine biosynthesis, reuptake, and degradation after TBI [30]. These alterations include variations in TH in the dopamine terminal, activities of monoamine oxidase and catechol-O-methyl transferase, and expression of dopamine transporter (DAT) affected after TBI. As previously mentioned, the TH stain decreased initially after TBI then increased gradually because of compensatory regrowth of the terminal after the TBI [30]. Futhermore, that the reuptake system was affected after TBI is another issue that caused the variation in the extracellular dopamine level [4].

Therefore, further studies are needed to evaluate the metabolism of dopamine, DA turnover, TH activity, DOPAC/DA and DA/TH in the nigrostriatal system after TBI to confirm whether such a compensatory mechanism exists after TBI.

On the other hand, previous reports on striatal dopamine content after TBI demonstrate no significant changes at 1 or 4 weeks post-injury [5]. As shown in Fig. 3C, the dopamine content in the striatum did not change significantly during the observation period. This lack of change may have been for two reasons. First of all, the dopamine biosynthesis pathway may have been affected by a surge of acute inflammatory cytokines after the TBI, which may have suppressed BH4, a cofactor of rate-limiting enzyme TH in the synthesis of dopamine; thus, the dopamine production may have been suppressed [45]. Second, the activities of monoamine oxidase and catechol-O-methyl transferase were affected after the TBI, which reduced the degradation of dopamine initially, and then the ratio of DOPAC or HVA to dopamine increased at the chronic stage (after 8 weeks, Fig. 3A). Further study is still needed to elucidate TBI-induced alterations of various synthesis and metabolizing enzymes for dopamine.

In addition, our HPLC data showed that the turnover rate of striatal dopamine tends to decrease initially in the 6-Pa-injured group and then increase after 8 weeks (p<0.05*) (Fig. 3A). The dopamine turnover rate tends to increase in the nucleus accumbens (NAc) initially in the 6-Pa-injured group and increase significantly at the chronic stage of injury (after 8 weeks) (Fig. 3B). However, the tissue dopamine concentration decreased in the 6-Pa-injured group 8 weeks after injury (Fig. 3C). Furthermore, the HPLC data showed significant changes in the dopamine turnover rate at 8 weeks in the 6-Pa group.

The dopamine transporter (DAT) plays a crucial role in determining the action of dopamine by regulating the reuptake of extracellular dopamine. Regional decreases in total DAT expression have been reported after CCI [4], [46]. Alterations in DAT expression suggest that improvements in cognition and neurobehavioral recovery reported in experimental [47]–[49] and clinical studies [11], [50].

The alterations in DAT expression can alter the kinetics of DA release, as demonstrated in DAT knockdown models [51], as can changes in DAT cellular localization [52]. Decreases in evoked DA overflow Vmax following CCI may be explained by either changes in expression or in membrane bound DAT associated with DAT trafficking [4], [46]. Given that a number of the current DA receptor agonist therapies act through a DAT mediated mechanism, it is necessary to fully understand the role of DAT changes in TBI in order to provide effective DA therapies.

Amandatine hydrochloride is a water soluble acid salt. Amantadine facilitates the release of dopamine, delays reuptake absorption in the presynapse, and increases the number of dopamine receptors in the post-synapse [53]. It also increases the extracellular DA levels in the striatum by inhibiting the re-uptake of DA and/or by blocking the channel in the N-methyl-D-aspartate (NMDA) receptor, which results in antagonism of NMDA receptor function and would be blocked by MK 801[54]–[56]. In this study, amantadine increased dopamine release as of 2 weeks after FPI (Figs. 1 and 2) and amantadine therapy also improved the cognitive deficit and motor behavioral impairment in injured animals as of 2 weeks after 6-Pa FPI. Improvements in the NOR and rotarod test results may have been due to dopamine release deficit after cerebral fluid percussion injury.

Comparing the dopamine release probability in the control, 6-Pa-injured, and 6-Pa-injured with amantadine treated groups (Fig. 5D) by examining peak concentrations elicited by single and multiple stimuli delivered at 25 Hz (see Materials and Methods) 8 weeks after injury showed a linear increase in DA concentration as a function of pulse number (Figs. 5D and E). 6-Pa-injured rat striatal slices demonstrated a significant reduction in DA concentration per pulse, relative to the control animals. Then amantadine treatment reversed the dopamine release probability (Fig. 5D: control rat slope: 38.0+4.6 nM/pulse (blue solid circle), 6-Pa-injured rat slope: 19.2+6.3 nM/pulse (red solid square) and 6-Pa+amantadine slope: 47.0±6.8 nM/pulse (gray open triangle), F = 4.550 (p = 0.021) of ANCOVA followed by SNK for multiple comparisons, control vs. 6-Pa-injured animal, p = 0.042*; control vs. 6-Pa+amantadine, p = 0.527; 6-Pa-injured animal vs. 6-Pa+amantadine, p = 0.007**). To determine the role of uptake in the regulation of frequency-dependent DA release in the control and 6-Pa-injured rats, the DAT inhibitor nomifensine was used. Nomifensine (5 µM) tended to increase (but only to a statistically insignificant degree) the frequency-dependent DA signal in the striata of the 6-Pa-injured animals but not those of the amantadine treated animals (Fig. 5E). Our data also indicated that the DAT function seems to be recovered at the chronic stage (8 weeks after injury), whereas nomifensine increased the probability of dopamine release at the brain slice. However, the effects of amantadine and nomifensine on the reuptake of the dopamine may also affect one another. Furthermore, the amantadine increase of the dopamine release may occur via presynaptic action to enhance DA release [54], [57] or inhibit DA uptake [58]. And in a report by Mizoguchi et al., the authors indicated that coadministration of nomifensine (10 mM, 120 min), an inhibitor of neuronal DA uptake, into the perfusion fluid attenuated the amantadine-induced increase in DA outflow [54]. The data from our dopamine releasing probability experiments showed a significant depression of the probability in 6-Pa injury with amantadine therapy under nomifensine infusion when compared with the data from 6-Pa-injured animals (Fig. 5E, the slope for nomifensine infusion, control: 101.5±2.5 - nM/pulse, 6-Pa: 169.2±2.3 nM/pulse; and 6-Pa injury with amantadine: 121.4±1.8 nM/pulse, F = 1.946 (p = 0.159) of ANCOVA followed by SNK for multiple comparisons, control vs. 6-Pa-injured animal, p = 0.058; control vs. 6-Pa+amantadine, p = 0.521, 6-Pa-injured animal vs. 6-Pa+amantadine, p = 0.375). We think that these data were still the result of the nomifensine affecting the enhancement of dopamine release induced by amantadine. So coadministration of nomifensine into the perfusion fluid attenuated the amantadine-induced increase in DA outflow, which is consistent with a previous report [54].

Moreover, the *tau* value increased in the 6-Pa-injured animals (Fig. 5A), but decreased gradually while the 6-Pa-injured animals received chronic amantadine therapy (Fig. 5B). These results may be due to the increase in dopamine level caused by the chronic amantadine therapy, and this increase may have induced the DAT function activation [59].

In 1994, Phillips et al. surveyed the motor balance and cognitive function after fluid percussion injury by using a rotarod test and Morris water maze. They also observed histological changes in the hippocampus using immunohistochemistry and electron microscopy to investigate the relationship between neuroexcitation and synaptic plasticity [60]. The rotarod test had been tested and was used as a tool to survey motor function deficits after fluid percussion injury [61].

The cognitive function and rotarod test were affected by 6-Pa fluid percussion injury. Then, the deficit of these tests was ameliorated by chronic amantadine pumping infusion therapy (for 7 weeks) and the improvement could be the result of dopamine release increase induced by amantadine and the recovery accelerated as early as 1 week after the start of the treatment. In this study, we performed FSCV on striatal brain slices at specific post-fluid percussion injury times to investigate dopamine release. The data shown here revealed severe suppression of dopamine release after 6-Pa injury and that this suppression could be reversed by chronic amantadine therapy. Thus, neuroprotection may be induced by amantadine; on the other hand, the fact that this increased dopamine release due to amantadine therapy could be suppressed by MK-801 was also shown in our study (Figs. 6A and B) [62]. However, this suppression did not block the increment dopamine releasing effect of amantadine completely, because the differences in the linear regression slopes of the control and amantadine+MK-801 groups were still significant (Fig. 6E, control vs. amantadine+MK 801, p<0.001***). So amantadine increases extracellular DA levels in the striatum not only by inhibiting the re-uptake of DA, but also by increasing releasing probability. These are associated with N-methyl-D-aspartate (NMDA) receptor, which may be competed and partial blocked by MK- 801, and such phenomena were also shown in our data (Figs. 6D and E) which indicated that an interaction between dopaminergic and glutamatergic neurotransmission is an important component in regulation of striatal dopamine level and were also consistent with that of a previous report [54], [63]. Furthermore, our data also indicates that in addition to its effects on NMDA receptors, the increase in dopamine release caused by the amantadine may mediate other mechanisms, a possibility which will require additional experiments for further investigation.

The behavioral improvement may thus result from two major mechanisms that were induced by amantadine infusion therapy; one is an increase in dopamine release and the other may result from neuroprotection due to NMDA inhibition. We do not claim, then, that the effect of amantadine in reducing behavioral deficits after FPI was only mediated by reversing the suppression of dopamine release. Our data show that the suppression of dopamine release after TBI was ameliorated by chronic infusion of the amantadine; this phenomenon may also be due to dopamine neuron protection after TBI as well as to inhibition of the reuptake of dopamine by amantadine infusion.

In summary, we performed a series of exams of dopamine release by using FSCV and behavioral exams after fluid percussion injury. Amantadine therapy with increased dopamine release had been proved in the series following time point in this study with the behavioral test in the meantime. Amantadine therapy improved the cognitive and motor deficit in 6-Pa-fluid percussion injury animal, which is compatible with the increase in dopamine release detected in our FSCV study in the treatment group.

Our findings suggest that chronic amantadine therapy accelerates recovery in cognitive and motor deficits after fluid percussion injury, which is consistent with the results from previous reports [6]–[8].

## Conclusion

In this study, we analyzed dopamine release as well as behavioral changes after fluid percussion injury with or without amantadine infusion pump treatment at acute (1 week), sub acute (2∼4 weeks), and chronic stages (6∼8 weeks) after injury. Severe suppression of both tonic and bursting dopamine release after fluid percussion injury was reversed by chronic amantadine therapy, which also improved behavioral impairments at the same time. The implications of dopamine-release suppression with regard to cognitive and motor learning deficits after TBI were addressed. The reversal of dopamine release changes and the improvement in behavioral deficits caused by chronic infusion of amantadine could provide value in chronic therapy for treating severe TBI.

## Supporting Information

Figure S1 **The diagram for the experimental protocol.** Each animal received its fluid percussion injury at the age of 6 weeks old, after which, according to the severity of the impaction injury, the animal would be placed into the mildly injured 2-Pa injury group (Group A) or the severely injured 6-Pa injury group (Group B). The severely injured animals then received either amantadine pump infusion therapy (Group C) or saline therapy (Group D). The group E is the control animal. The infusion pumps were implanted into group C and D animals at 3 days post-injury. The FSCV study was performed on Groups A, B, C, and E at 1, 2, 4, 6, and 8 weeks post-injury. The rotarod test was performed by Groups B, C, D, and E once per week beginning at 1 week post-injury. The NOR test was performed for Groups B, C, D, and E at 1, 2, 4, 6, and 8 weeks. The HPLC test was performed on Groups A, B, and E at 2 hr, 1 day, and 1, 2, and 8 weeks post-injury. (Note: *indicates p<0.05; **indicates p<0.01; and ***indicates p<0.001)
(TIF)

Figure S2 **The IT/CV curve of voltammetry at sequential time points; 1 (A–D, 7 days after 6-Pa fluid percussion injury), 2 (E–H, 14 days after 6-Pa fluid percussion injury), 4 (I–L, 4 weeks after 6-Pa fluid percussion injury), 6 (M–P, 6 weeks after 6-Pa fluid percussion injury), and 8 weeks (Q–T, 8 weeks after 6-Pa fluid percussion injury) after injury of the control (solid line), 6-Pa-injury (black dotted line), and 6-Pa-injury with amantadine therapy animals (gray dotted line).** (Note: *indicates p<0.05; **indicates p<0.01; and ***indicates p<0.001)
(TIF)
