# Supplementary figures and images for: Amantadine Ameliorates Dopamine-Releasing Deficits and Behavioral Deficits in Rats after Fluid Percussion Injury
Source: PLoS One. 2014 Jan 30;9(1):e86354. doi: 10.1371/journal.pone.0086354 (PMC3907421; doi:10.1371/journal.pone.0086354)

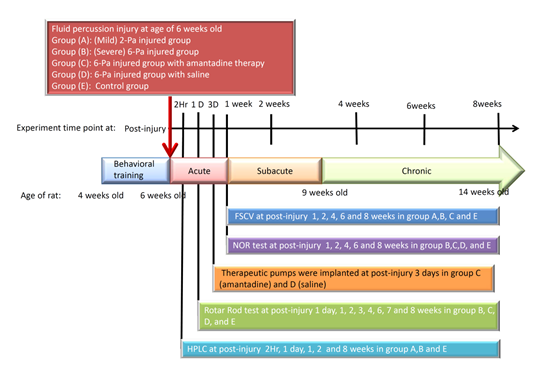

Supplement: Figure S1 — The diagram for the experimental protocol. Each animal received its fluid percussion injury at the age of 6 weeks old, after which, according to the severity of the impaction injury, the animal would be placed into the mildly injured 2-Pa injury group (Group A) or the severely injured 6-Pa injury group (Group B). The severely injured animals then received either amantadine pump infusion therapy (Group C) or saline therapy (Group D). The group E is the control animal. The infusion pumps were implanted into group C and D animals at 3 days post-injury. The FSCV study was performed on Groups A, B, C, and E at 1, 2, 4, 6, and 8 weeks post-injury. The rotarod test was performed by Groups B, C, D, and E once per week beginning at 1 week post-injury. The NOR test was performed for Groups B, C, D, and E at 1, 2, 4, 6, and 8 weeks. The HPLC test was performed on Groups A, B, and E at 2 hr, 1 day, and 1, 2, and 8 weeks post-injury. (Note: *indicates p<0.05; **indicates p<0.01; and ***indicates p<0.001) (TIF) [file pone.0086354.s001.tif]

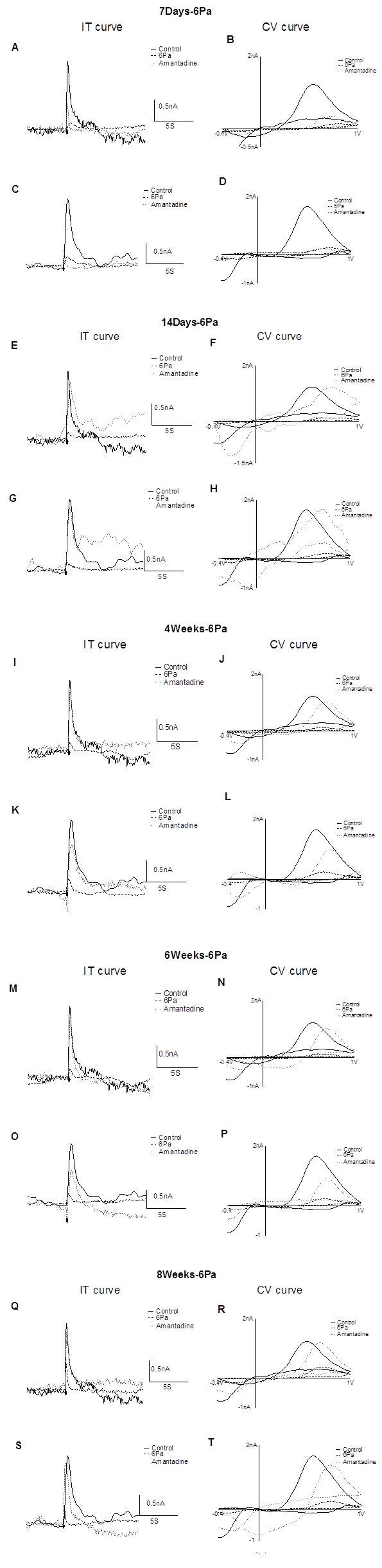

Supplement: Figure S2 — The IT/CV curve of voltammetry at sequential time points; 1 (A–D, 7 days after 6-Pa fluid percussion injury), 2 (E–H, 14 days after 6-Pa fluid percussion injury), 4 (I–L, 4 weeks after 6-Pa fluid percussion injury), 6 (M–P, 6 weeks after 6-Pa fluid percussion injury), and 8 weeks (Q–T, 8 weeks after 6-Pa fluid percussion injury) after injury of the control (solid line), 6-Pa-injury (black dotted line), and 6-Pa-injury with amantadine therapy animals (gray dotted line). (Note: *indicates p<0.05; **indicates p<0.01; and ***indicates p<0.001) (TIF) [file pone.0086354.s002.tif]
